# Ovine endometrial estrogen receptor expression is altered following PG‐600 administration

**DOI:** 10.1002/vms3.1119

**Published:** 2023-03-15

**Authors:** Hayder Mohammed Hassan Habeeb, Logan Kleditz, Timothy Hazzard, Cecily Bishop, Fred Stormshak, Michelle Anne Kutzler

**Affiliations:** ^1^ Department of Animal Production Al‐Qasim Green University Babylon Iraq; ^2^ College of Engineering Oregon State University Corvallis Oregon USA; ^3^ Department of Animal and Rangeland Sciences Oregon State University Corvallis Oregon USA; ^4^ Division of Reproductive and Developmental Sciences, Oregon National Primate Research Center Oregon Health & Science University Beaverton Oregon USA

**Keywords:** endometrium, estradiol, ewes, estrogen receptor, progesterone

## Abstract

**Background:**

Dysregulation of the estrogen receptor 1 (ESR1) expression during the establishment of pregnancy could contribute to reduce fertility reported in ewes treated with PG‐600.

**Objectives:**

The objective of this study was to evaluate the effect of treatment with PG‐600 on ESR1 expression in the ovine endometrium during early diestrus.

**Methods:**

Polypay ewes (*n* = 24) that had exhibited an oestrous cycle of normal duration (16–18 days) were treated with intravaginal progesterone‐releasing device (CIDR) for 9 days. Cloprostenol was administrated two days before CIDR withdrawal. On the day of CIDR withdrawal (day 0), ewes received a 5 mL intramuscular injection of PG‐600 (400 IU equine chorionic gonadotropin and 200 IU human chorionic gonadotropin) or saline. Blood samples were collected before treatment and on the day of tissue collection to determine serum estradiol‐17β and progesterone concentrations using radioimmunoassay. On days 4 and 7, six ewes from each treatment group were anaesthetised and a laparotomy was performed to obtain intercaruncular endometrial samples. Endometrial samples were collected ipsilateral to the ovary bearing the highest number of corpora lutea. An estradiol exchange assay was used to determine ESR1 concentration.

**Results:**

Estradiol concentrations did not differ by time or treatment, but progesterone concentrations were significantly higher in the PG‐600‐treated group on day 7 despite a similar number of corpora lutea. Endometrial ESR1 concentration was significantly reduced on day 7 in the PG‐600 group.

**Conclusions:**

In conclusion, although progesterone was higher than controls on day 7, ESR1 did not differ from controls suggesting that treatment with PG600 is unlikely to impair reproductive potential.

## Graphical abstract

PG‐600 administration may indirectly cause downregulation of the endometrial ESR on day 7 of the ovine oestrous cycle through an enhanced production of progesterone by the corpora lutea. There was no effect of treatment on serum concentrations of estradiol, although estradiol concentration was higher on days 4 and 7 of the oestrous cycle than what has been previously reported in ewes.

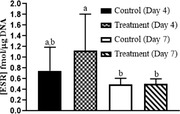

## 1 INTRODUCTION

Sheep are short‐day seasonal breeders, which means they ovulate during periods of decreasing daylight and do not naturally ovulate during periods of increasing daylight. Ewe productivity can be increased by employing hormonal methods, which induce ovulation outside of the breeding season (Umberger et al., 1994). PG‐600 is a single dose injectable product labelled for use in swine but can be used off‐label for estrus induction in ewes (Cross et al., 2019; Safranski et al., 1992). PG‐600 (Intervet/Merck Animal Health, Madison, NJ, USA) contains 80 IU/mL of equine chorionic gonadotropin (eCG) and 40 IU/mL of human chorionic gonadotropin (hCG). Our laboratory reported that PG‐600 administered to cycling ewes resulted in elevated serum estradiol‐17β concentrations within the first day after administration of PG‐600 and reduced pregnancy rates (Habeeb et al., 2019).

Like follicle stimulating hormone (FSH), eCG binds to the follicle stimulating hormone receptor (FSHR) (Licht et al., 1979). Ovarian FSHRs are present in granulosa cells of small and preovulatory follicles, which induce follicular growth and development (Tisdall et al., 1995) and thus, secretion of estradiol. In women, elevated serum estradiol can contribute to the embryo mortality experienced in early pregnancy (Kolibianakis et al., 2003).

Elevated serum estradiol‐17β concentration can increase ovine endometrial estrogen receptor (ESR1) concentration (Spencer et al., 1995). Reduction in ESR1 during early to mid‐diestrus is presumed to be due to rising concentrations of circulating progesterone (Karsch et al., 1980). In cycling ewes, endometrium ESR1 mRNA and protein expression is highest on day 1 of the oestrous cycle, declines between days 1 and 6, and then increases between days 11 and 15 (Spencer et al., 1995). Interestingly, estradiol upregulates endometrial ESR1 mRNA by stabilising the message (Mitchell & Ing, 2003). The increase in ESR1 expression at the end of the cycle coincides with an increase in oxytocin receptor expression, which allows oxytocin to induce uterine release of luteolytic prostaglandin‐F pulses (Spencer et al., 1995). However, controlled experiments using pure antiestrogens or gene knockdown technology have not been conducted to confirm that endometrial ESR1 induction is required for oxytocin gene expression and luteolysis. Dysregulation of the expression of ESR1 during the establishment of pregnancy could contribute to the reduced fertility reported in ewes treated with PG‐600 (Lunstra & Christenson, 1981).

The objective of this research was to determine the effect of PG‐600 treatment on endometrial ESR1 expression and serum concentrations of estradiol and progesterone on days 4 and 7 of the ewes oestrous cycle. We hypothesised that administration of PG‐600 would alter ESR1 expression in the endometrium compared to saline‐treated controls.

## 2 MATERIALS AND METHODS

### 2.1 Animals

During early fall (September in the Northern Hemisphere) cycling Polypay ewes (*n* = 24), ages 2–7 (3.1 ± 1.5 years), were administered an intravaginal progesterone‐releasing device (Eazi‐Breed® CIDR, Zoetis, Kalamazoo, MI, USA) for 9 days. Forty‐eight hours before progesterone (P4) withdrawal (d2), ewes were treated with intramuscular cloprostenol sodium (125 μg, Estrumate®, Intervet/Merck Animal Health, Madison, NJ, USA). On the day of P4 withdrawal (day 0), ewes were divided randomly into a two‐by‐two factorial arrangement of treatment groups consisting of exposure to PG600 (3.3 ± 1.6 years; *n* = 12) and control (3.0 ± 1.4 years; *n* = 12), with two separate days postexposure in the luteal phase for analyses. PG‐600 groups received a single intramuscular injection of PG‐600 (400 IU eCG and 200 IU hCG in 5 mL total volume), while controls received vehicle only (5 mL saline). Endometrial tissue collection was performed either on day 4 (early luteal) or 7 (mid‐luteal) posttreatment, resulting in final treatment groups of 4 and 7 days post‐PG‐600 (*n* = 6 ewes/group).

Immediately following progesterone withdrawal (day 0), jugular venous blood samples were collected prior to administration of PG‐600 or saline. Additionally, blood samples were collected at the time of surgery. Blood samples were centrifuged at 1620 × *g* after overnight storage at 4°C. Sera were separated and stored at −20°C until analysed for progesterone and estradiol‐17β. Serum progesterone and estradiol‐17β concentrations were determined using radioimmunoassay.

### 2.2 Estradiol‐17β and progesterone concentration

Serum concentrations of estradiol‐17β and progesterone were measured by the Endocrine Technologies Core at the Oregon National Primate Research Center (ONPRC)^b^ using established radioimmunoassays following chromatographic separation (Rasmussen et al., 1984; Reddy et al., 2014; Roselli et al., 2011). Briefly, serum samples were extracted with 6 mL diethyl ether, dried under forced air, and redissolved in 200 μL of column solvent (hexane:benzene:methanol = 62:20:13). The samples were then added to 1×6 cm all‐glass columns containing 1.0 g Sephadex LH‐20 for chromatographic separation of steroids. The fractions containing estradiol‐17β and progesterone were collected, dried under forced air, reconstituted, and subjected to specific radioimmunoassay (RIA) for each hormone (custom rabbit antibodies: anti‐progesterone, clone Surve #1 at 1:9090 and anti‐estradiol clone #244, at 1:4167). Hormone values were corrected for extraction losses determined by radioactive trace recovery, performed at the same time as sample extraction (^3^H‐estradiol‐17β and ^3^H‐progesterone, American Radiolabeled Chemicals, St. Louis, MO, USA). Estradiol and progesterone recovery was 90.0% for estradiol‐17β and 101% for progesterone. The intraassay coefficient of variation (CV) for estradiol‐17β was 6.9% and for progesterone was 6.5%. The interassay CV was less than 15% for both estradiol‐17β and progesterone. The lowest level of detection for both estradiol‐17β and progesterone assays was 5 pg/mL.

### 2.3 Endometrial tissue collection

Ewes were anaesthetised with intravenous diazepam (7.5 mg) and ketamine (300 mg) and anaesthesia was maintained with isoflurane for 1–2 h. The ventral midline was clipped and surgically prepared for a laparotomy. Number of corpora lutea was counted for each ovary and for each animal. An incision was made into the lumen of the uterine horn ipsilateral to the ovary with the greatest number of developing corpora lutea. The horn ipsilateral to the ovary with the greatest number of corpora lutea was selected for consistency among ewes that may have only ovulated on the left or the right ovary. Intercaruncular endometrial tissue (2 g) was excised using Metzenbaum scissors. Because the focus of this research was to evaluate receptor expression in endometrial glandular tissue, intercaruncular endometrial tissue was collected instead of caruncular tissue. Three‐fourths (1.5 g) of endometrial tissue was placed into oxygenated Dulbecco's Modified Eagle media (phenol‐free). Internal surgical wounds were closed using a simple continuous pattern with 2‐0 monofilament polydiaxanon on the uterine horn, and #1 coated braided polyglycolic acid suture on the abdominal wall and subcutaneous tissue (Henry Schein, Dublin, OH, USA). The skin was closed with polyamide non‐absorbable suture (Braunamid®, Melsungen, Germany) in a Ford interlocking pattern. Postoperative analgesia was accomplished using a fentanyl patch (50 μg, Watson, Actavis, Libertyville, IL, USA) for 3 days and intramuscular flunixin meglumine (100 mg/50 kg body weight, Merck Animal Health, Madison, NJ, USA) once daily for 3 days.

### 2.4 Estrogen exchange assay

An estradiol exchange assay was used to determine ESR1 concentration as previously described by Koligian and Stormshak (1977a). Briefly, 1.5 g of endometrial tissue was placed directly into oxygenated (95% O_2_–5% CO_2_) media (DMEM/F‐12, Life Technology, Carlsbad, CA, USA) and then homogenised in cold 0.01 M Tris HCL–0.0015 M EDTA (TE) buffer (pH 7.4) at 10 s intervals with intermittent cooling until thoroughly homogenised. Homogenised tissue was centrifuged at 800 × *g* for 10 min and washed three times with 2 mL TE buffer. The pellet was vortexed for few seconds in 50 mM Tris buffer (pH 7.3). Aliquots of the nuclear pellet were resuspended with either 2 × 10^−6^ M [^3^H] estradiol or 2 × 10^−6^ M [^3^H] estradiol with 200 × 10^−6^ M diethylstilbestrol (DES, Sigma‐Aldrich, St. Louis, MO, USA). An additional aliquot was washed with 0.3 M perchloric acid (PCA, Macron Fine Chemicals, Valley, PA, USA) for DNA determination (see below). Estradiol exchange activity occurred after a 30‐min incubation at 37°C followed by a 10‐min incubation at 4°C. Following cooling, 50 mM Tris HCL buffer was added to all tubes and tubes were centrifuged at 800 × *g* for 5 min. Supernatants were decanted after two consecutive washes and the nuclear pellets were incubated overnight in 2 mL of absolute ethanol. The ethanol extract (2 mL) was decanted into scintillation fluid (6 mL; MP Biomedicals, Solon, OH, USA) and the radioactivity of each sample was counted (Beckman Model LS‐6000‐TA Liquid Scintillator, Philadelphia, USA). The difference in counts per minute (CPM) between samples exposed to only [^3^H] estradiol (total binding) and those samples exposed to [^3^H] estradiol and 100‐fold DES (nonspecific binding) was taken to represent specific binding of the estradiol.

To determine tissue level of DNA, calf thymus DNA (Sigma‐Aldrich, Louis, MO, USA) was used as a standard (0 to 500 μg). Endometrial samples (100 mg) were centrifuged, and pellets were washed with cold 0.3 M PCA three times at 1620 × *g* for 5 min each. Bovine serum albumin buffer (BSA, 0.1%, Sigma‐Aldrich, Louis, MO, USA) was added to the standards. Cold 0.5 M PCA (1:1) was added to both standards and samples. Standards and samples were incubated on ice for 15 min. Standards and samples were centrifuged at 1620 × *g* for 10 min and the pellets were resuspended with 2 mL room temperature 0.5 M PCA. Standards and samples were incubated at 90°C for 30 min, cooled for 30 min, and then centrifuged at 1620 × *g* for 5 min. Standards and samples were decanted into a clean tube and 1 mL diphenylamine (Sigma‐Aldrich, Louis, MO) was added to all tubes, incubated in dark for 24 h at room temperature, and then read spectrophotometrically at 600 nm (Thermo Spectronic, Rochester, NY, USA) to determine the DNA content (Bishop & Stormshak, 2006; Burton, 1956; Koligian & Stormshak, 1977a, 1977b). Sample level of DNA was used to express concentration of estrogen receptor (ESR1) in fmol/μg DNA.

### 2.5 Statistical analyses

Discrete variables for this study (effect of PG‐600 administration, endometrial ESR1 concentration and number of corpora lutea) were analysed by two‐way analysis of variance with factors of treatment (PG‐600 or control) and day of tissue collection (4 vs. 7), and interaction between treatment and day as appropriate for the experimental design (GraphPad Prism 9, San Diego, CA). Two‐way analysis of variance with repeated measures was performed to determine the effect of PG‐600 administration, time, and interaction between PG‐600 administration and time on estradiol‐17β and progesterone concentration. Significance was defined as *p* < 0.05. Endometrial ESR1 expression as well as serum estradiol‐17β and progesterone concentration were reported as a mean ± standard deviation.

## 3 RESULTS

**FIGURE 1:**
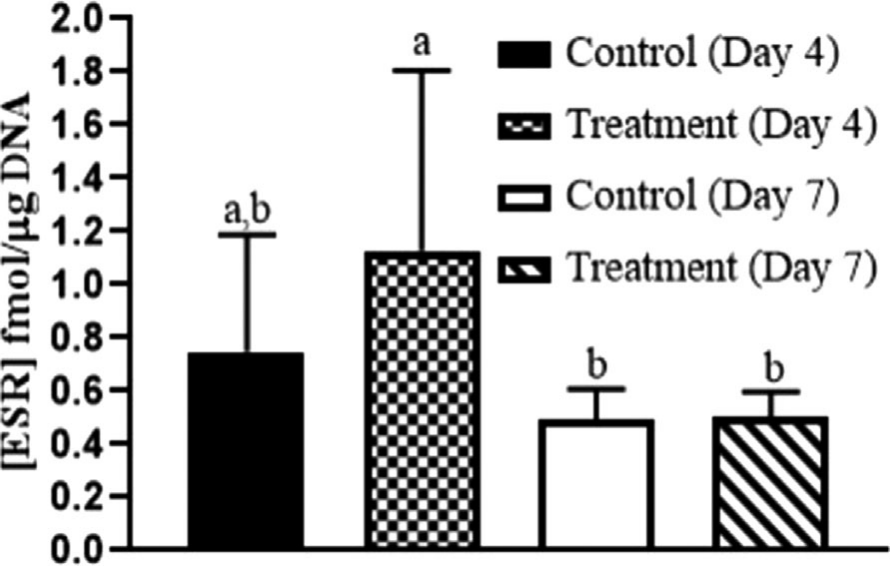
Mean (± SD) intercaruncular endometrial estrogen receptor 1 (ESR1) concentration at 4 and 7 days after treatment of cycling ewes with 5 mL of PG‐600 or saline. Intercaruncular endometrial ESR1 concentration did not differ with administration of PG‐600 but did differ between day 4 and day 7 in the PG‐600‐treated group (Day, *p* < 0.01). Comparisons between values are the results of pairwise analyses. Values with different superscript letters (^ab^) are significantly different (*p* < 0.01).

**FIGURE 2:**
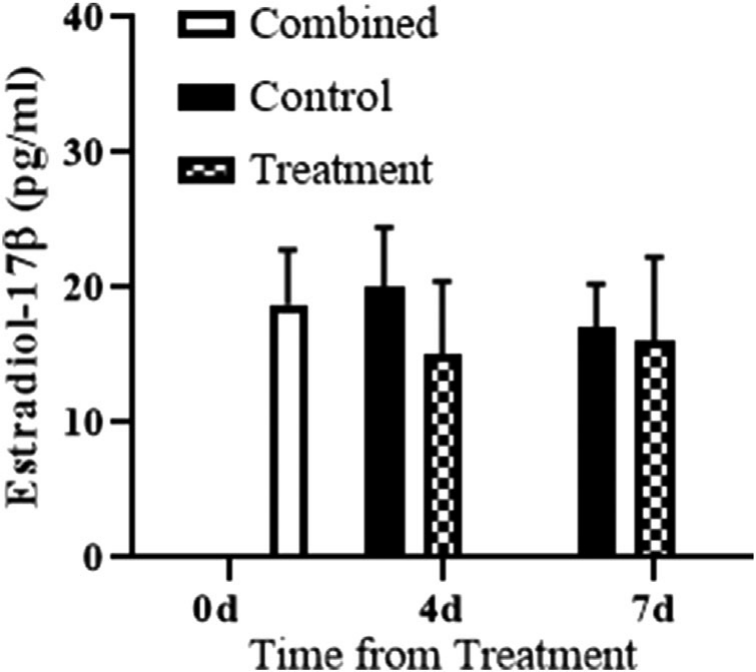
Mean (± SD) serum estradiol‐17β concentration was measured before treatment (0 day) and at the time of endometrial sample collection (either 4 or 7 days after treatment of ewes with 5 mL of saline (control) or PG‐600 (treatment).

**FIGURE 3:**
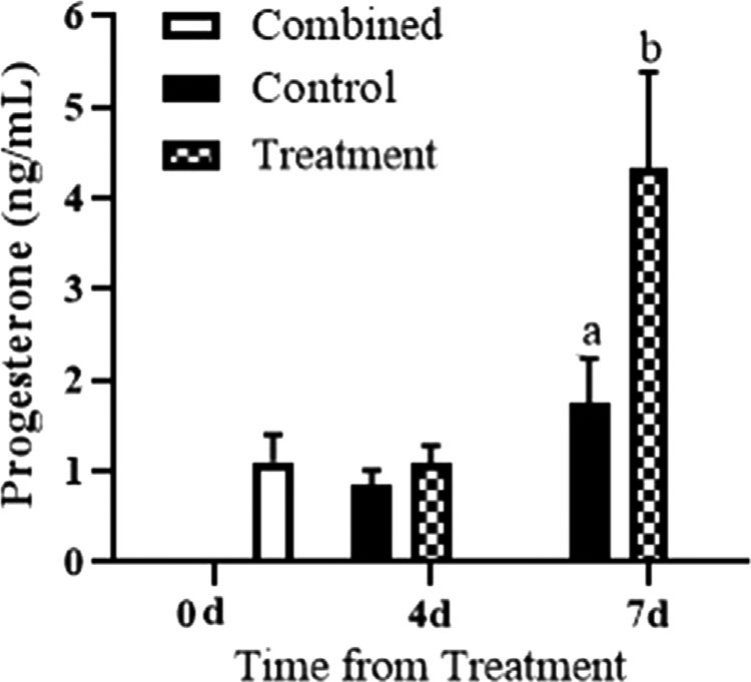
Mean (± SD) serum progesterone concentration immediately before treatment (0 day) and at the time of endometrial sample collection (either 4 or 7 days after treatment in ewes treated with 5 mL of saline (control) or PG‐600 (treatment). Serum progesterone concentration was similar in control and PG‐600‐treated ewes on day 4 and increased in both groups on day 7 with concentration in treated ewes being markedly greater than in controls (Treatments by Day interaction, *p* < 0.0001. Values with different superscript letters (^ab^) are significantly different (*p* < 0.01).

**FIGURE 4:**
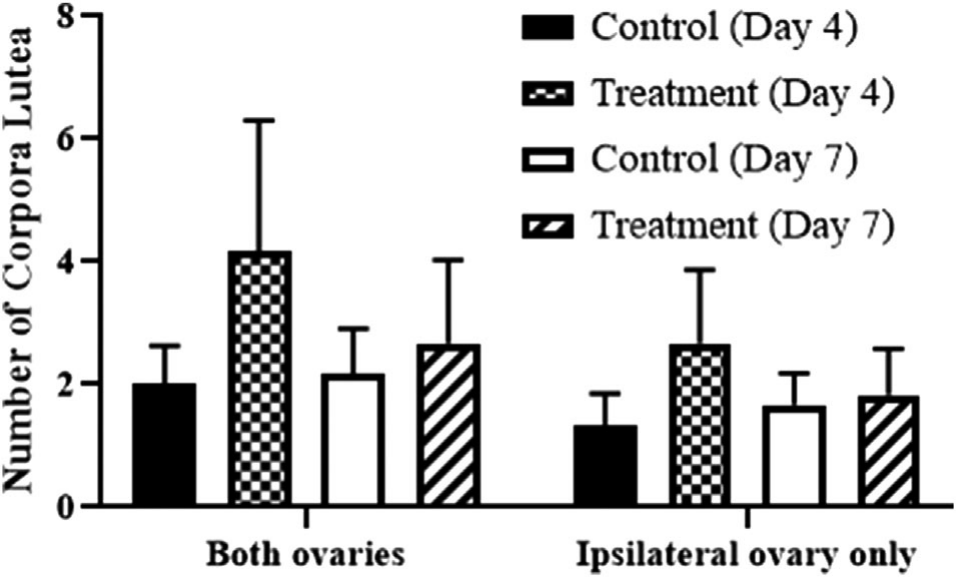
Mean (± SD) of total number of corpora lutea on both ovaries and number ipsilateral to the uterine horn where endometrial tissue was analysed for estrogen receptor expression.

Intercaruncular endometrial ESR1 concentrations were greater in PG‐600‐treated ewes on day 4 and were decreased on day 7 (Day, *p* < 0.01, Figure 1). However, there was no change in ESR1 between days 4 and 7 in control‐treated ewes (Figure 1). Serum estradiol‐17β concentration did not differ with PG‐600 administration or day of diestrus, or their interaction (Treatment, Day, Treatment by Day, all *p* > 0.05, Figure 2). Serum progesterone concentration was similar in control and PG‐600‐treated ewes on day 4 and increased in both groups on day 7 with concentration in treated ewes being markedly greater than in controls (Treatments by Day interaction, *p* < 0.0001, Figure 3). The number of corpora lutea ipsilateral to the endometrial tissue sampled uterine horn and the total number of corpora lutea per ewe did not differ by PG‐600 administration, day of diestrus, or their interaction (Treatment, Day, Treatment by Day, all *p* > 0.50, Figure 4). Serum progesterone concentration on day 0 reflects the status of the regressing corpora lutea in the ewes.

## 4 DISCUSSION

Equine chorionic gonadotropin (eCG) is a gonadotropin synthesised by pregnant mares with prolonged FSH‐like properties (Lunenfeld, 2011). In the United States, eCG alone is not commercially available but comes in a compounded form (PG‐600) that contains eCG (80 IU/mL) and human chorionic gonadotropin (hCG, 40 IU/mL) (Estienne & Harper, 2001). Although this product is labelled for use in swine, US sheep producers have used it for many years for oestrous synchronisation (Cross et al., 2019; Safranski et al., 1992). However, the recommended dose (5 mL) is known to overstimulate the ovine ovary resulting in abnormally large follicles with increased estradiol‐17β concentrations at the time of ovulation (Safranski et al., 1992). In addition, fertilisation rates (as determined by the number of recovered embryos following oviductal flushing relative to the number of corpora lutea) in ewes treated with eCG both during the breeding season and out of season were reduced compared to fertilisation rates of untreated ewes during the breeding season (Lunstra & Christenson, 1981). Additional research is needed to determine an effective lower dose of PG‐600 that does not overstimulate the ovaries or reduce fertilisation rates.

In pregnant ewes, the conceptus trophoblast produces interferon tau (IFN tau) that acts on the endometrium to inhibit transcription of the ER alpha gene directly and the oxytocin receptor gene indirectly to abrogate development of the endometrial luteolytic mechanism (Spencer et al., 1995). In the current study, serum estradiol‐17β concentrations did not differ by day of diestrus, administration of PG‐600, or their interaction but were higher on days 4 and 7 of the oestrous cycle than what has been previously reported in ewes (Karsch et al., 1980). On the other hand, endometrial ESR1 concentration was significantly reduced on day 7 after administration of PG‐600. It is important to note that serum progesterone concentrations were significantly higher on day 7 compared to day 0 in both the control and PG‐600‐treated ewes in this study. Using ovariectomised ewes treated with progesterone, Koligian and Stormshak (1977b) found that progesterone downregulated nuclear ESR1. This was supported by a similar study in which a 1.89‐fold decline in endometrial ESR1 concentration between day 6 and day 10 of the ovine oestrous cycle was postulated to be due to an increase in progesterone (Koligian & Stormshak, 1977a; Tasende et al., 2002). Thus, elevated serum progesterone concentrations in the current study may be due to the long‐lasting effects of the hCG on the ovine corpora lutea (Coleson et al., 2015).

The influence of the number of corpora lutea on progesterone concentrations is contradictory. In sows, there is no relationship between progesterone concentration measured from the ipsilateral utero‐ovarian vein and the number of CLs (Kotwica et al., 1984). In addition, the number of corpora lutea is not significantly correlated with circulating plasma progesterone concentrations in mice (Simon et al., 1978) and rabbits. However, goats with multiple corpora lutea had significantly higher circulating progesterone concentrations from days 7 to 30 of pregnancy than goats with only one corpus luteum (Jarrell & Dziuk, 1991). In ewes, the influence of the number of corpora lutea on progesterone concentrations is not known but as the number of corpora lutea increase, the mass of each individual corpus luteum decreases and this effect is not a result of crowding within the ovarian parenchyma (Ginther, 1971).

With respect to responses following gonadotropin administration, heifers receiving eCG twice 4 days apart with the initial administration being two days after GnRH administration had a significantly higher number of corpora lutea and greater progesterone concentration compared to the single eCG‐treated group (Mahdavi‐Roshan et al., 2020). Ewes administered eCG for 3 days following intravaginal progesterone removal had more corpora lutea and higher progesterone concentration compared to ewes treated with eCG for 0, 1 or 2 days (Shabankareh et al., 2012). In addition, ewes administered eCG for 4 days following mating had more corpora lutea and higher progesterone concentration compared to the control group (Coleson et al., 2015).

In conclusion, this is the first report to examine the effect of PG‐600 on ovine uterine ESR1 concentrations. Because PG‐600 use in ewe has been associated with reduced fertility (Cross et al., 2019; Habeeb et al., 2019; Lunstra & Christenson, 1981), the results presented in this report demonstrate that uterine changes in ESR1 during the first week postoestrus after treatment are comparable to those found in normal cycling ewes. In conclusion, it appears unlikely that any of the observed changes in uterine ESR1 and estradiol and progesterone production after PG‐600 treatment of ewes would impair their reproductive potential.

## AUTHOR CONTRIBUTIONS

Hayder Habeeb contributed to the conceptualisation, data curation, formal analysis, funding acquisition, investigation, methodology, project administration, supervision, validation, visualisation, writing – original draft, writing – review & editing. **Logan Makenzie Kleditz** contributed to funding acquisition, investigation, writing – review & editing. **Timothy Hazzard** contributed to the resources, writing – original draft. **Cecily Bishop** contributed to the data curation, investigation, methodology, resources, supervision, visualisation, writing – original draft. **Fredrick Stormshak** contributed to the conceptualisation, formal analysis, methodology, resources, validation, visualisation, writing – original draft, writing – review & editing. **Michelle Kutzler** contributed to the conceptualisation, formal analysis, funding acquisition, methodology, resources, supervision, validation, writing – original draft, writing – review & editing.

## CONFLICT OF INTEREST STATEMENT

The authors disclose there were no actual or potential conflicts of interest regarding the research that affected their ability to objectively present or review the research or data.

### ETHIC STATEMENT

The authors confirm that the ethical policies of the journal of veterinary medicine and science, as found in the journal's guidelines, have been adhered to and All animal experiments were approved by the Oregon State University Institutional Animal Care and Use Committee (protocol #5036).

### PEER REVIEW

The peer review history for this article is available at https://publons.com/publon/10.1002/vms3.1119.

## Data Availability

The data that supports the findings of this study are available from the corresponding author upon reasonable request.
